# AdipoR1 promotes pathogenic Th17 differentiation by regulating mitochondrial function through FUNDC1

**DOI:** 10.7555/JBR.38.20240244

**Published:** 2024-11-07

**Authors:** Hui Wang, Qian Zhang, Yuankai Sun, Wenfeng Tan, Miaojia Zhang

**Affiliations:** Department of Rheumatology, the First Affiliated Hospital of Nanjing Medical University, Nanjing, Jiangsu 210029, China

**Keywords:** AdipoR1, pTh17, FUNDC1, mitochondrial function

## Abstract

Adiponectin receptor 1 (*Adipor1*) deficiency has been shown to inhibit Th17 cell differentiation and reduce joint inflammation and bone erosion in antigen-induced arthritis mice. Additional emerging evidence indicates that Th17 cells may differentiate into pathogenic (pTh17) and non-pathogenic (npTh17) cells, with the pTh17 cells playing a crucial role in numerous autoimmune and inflammatory conditions. In the current study, we found that *Adipor1* deficiency inhibited pTh17 differentiation *in vitro* and induced mitochondrial dysfunction in pTh17 cells. RNA sequencing demonstrated a significant increase in the expression levels of *Fundc1*, a gene related to mitochondrial function, in *Adipor1*-deficient CD4^+^ T cells. *Fundc1* knockdown in *Adipor1*-deficient CD4^+^ T cells partially reversed the effects of *Adipor1* deficiency on mitochondrial function and pTh17 differentiation. In conclusion, the current study demonstrated a novel role of *Adipor1* in regulating mitochondrial function *via* *Fundc1* to promote pTh17 cell differentiation, providing some insight into potential therapeutic targets for autoimmune and inflammatory diseases.

## Introduction

A subpopulation of CD4^+^ T cells, known as T helper 17 (Th17) cells, express the retinoid-related orphan receptor gamma-t (RORγt) and produce cytokines, such as interleukin-17 (IL-17) and IL-22^[1–2]^. Th17 cell responses are implicated in the pathogenesis of various autoimmune diseases, such as rheumatoid arthritis (RA), multiple sclerosis, inflammatory bowel disease, and psoriasis^[3]^. Recent studies have revealed the heterogeneity of Th17 cells, which may differentiate into two subpopulations based on their pathogenicity: pathogenic Th17 (pTh17) cells and non-pathogenic Th17 (npTh17) cells^[4]^. The *in vitro* differentiation of both pTh17 and npTh17 cells occurs under various cytokine conditions^[5]^. pTh17 cell differentiation is driven by IL-6, IL-23, and IL-1β^[6]^, while transforming growth factor-β1 (TGF-β1) and IL-6 promote npTh17 cell differentiation^[7–8]^. Although both pTh17 and npTh17 cells share the Th17 markers, such as IL-17A and RORγt, they exhibit distinct transcriptional profiles that determine their roles in either promoting immune disorders or maintaining immune balance^[5,9]^.

pTh17 cells are rich in pro-inflammatory cytokines such as granulocyte-macrophage colony-stimulating factor (GM-CSF) and IL-23 receptor (IL-23R), but lack immune regulatory components such as IL-10 and CD5 molecule-like (CD5L). Conversely, npTh17 cells exhibit minimal levels of GM-CSF and IL-23R, while showing elevated levels of IL-10 and CD5L, which support tissue stability^[6–7,10–12]^. pTh17 cells may also be distinguished from npTh17 cells by transcription factors (*Stat4* and *Tbx21*)^[6]^. RA patients have been found to have a higher percentage of pTh17 cells in some studies. These cells may generate a significant amount of inflammatory cytokines, exhibit resistance to apoptosis, and demonstrate a lack of responsiveness to treatment. Moreover, pTh17 cells are difficult to suppress by Treg cells^[4,13]^.

Adiponectin, a cytokine released by adipose tissues, plays a critical role in immune responses and metabolic processes. The functions of adiponectin are carried out through its interaction with its receptors^[14]^. One of our previous studies showed that adiponectin and adiponectin receptor 1 (AdipoR1) were highly expressed in RA synovial tissues^[15]^. Adiponectin promotes the transformation of naïve CD4^+^ T cells into Th17 cells, contributing to synovial membrane inflammation and bone erosion in RA patients^[16]^. Conversely, *Adipor1* deficiency inhibits Th17 cell differentiation and reduces joint inflammation and bone erosion in antigen-induced arthritis mice^[17]^, suggesting that AdipoR1 may be involved in the inflammatory process of RA by regulating Th17 differentiation. However, whether AdipoR1 is involved in regulating pTh17 differentiation and the underlying molecular mechanisms remains unclear.

Mitochondria and mitochondrial membrane remodeling influence complex cell signaling events, including those regulating cell pluripotency, division, differentiation, aging, and apoptosis^[18]^. It has been shown that defects in electron transport chain complexes, impairment of oxidative phosphorylation, and altered mitochondrial morphology all play key roles in the differentiation of pTh17 cells^[19]^; however, attenuated pTh17 cell function has been related to the downregulated expression of mitochondrial function-related genes^[19–20]^.

FUN14 domain-containing 1 (FUNDC1) is a novel mitochondrial membrane protein that acts as a receptor for hypoxia-induced mitochondrial autophagy^[21]^. FUNDC1-mediated mitochondrial autophagy selectively removes dysfunctional mitochondria, which in turn reduces damage to cells and tissues^[22–23]^. FUNDC1 also plays a crucial role in mitochondria-associated membranes (MAMs), the interface between mitochondria and the endoplasmic reticulum, by promoting the stabilization of MAMs and facilitating the transfer of endoplasmic reticulum Ca^2+^ to mitochondria and the cytoplasm^[24]^.

In the current study, we investigated the effects of *Adipor1* knockout on pTh17 cell differentiation and mitochondrial function. Because FUNDC1 and mitochondrial function are closely related, we further determined the role of *Adipor1* in pTh17 cell differentiation and mitochondrial function in the context of FUNDC1 expression.

## Materials and methods

### Mice

As detailed in the reference^[17]^, we created *Adipor1*^fl/fl^*Cd4-Cre* conditional knockout (CKO) mice. Cre-negative littermates are referred to as wild-type (WT) controls. All mice used were of the C57BL/6 genetic background. We used only male mice aged between 6 and 8 weeks, maintained under germ-free conditions. All animal experiments were approved by the Institutional Animal Care and Use Committee of Nanjing Medical University, Nanjing, Jiangsu, China (IACUC-2013090101).

### Isolation and differentiation of T cells *in vitro*

In the Th17 differentiation experiments performed *in vitro*, we extracted naïve CD4^+^ T cells (CD4^+^CD62L^+^ T cells) from the spleen, and refined the single-cell suspensions through negative selection using magnetic beads (Cat. #19765A, Stemcell, Vancouver, BC, Canada). We pretreated the 96-well plates with 50 μL phosphate-buffered solution (PBS) containing 10 μg/mL anti-CD3e mAb (eBioscience, San Diego, CA, USA) and 3 μg/mL anti-CD28 mAb (eBioscience) at 4 ℃ for 16 h. We then added 200000 naïve CD4^+^ T cells to 200 μL of T cell medium (RPMI with 10% FBS). To differentiate naïve CD4^+^ T cells into npTh17 cells, the culture medium was supplemented with 20 ng/mL IL-6 (Peprotech, Cranbury, NJ, USA) and 0.5 ng/mL TGF-β (Peprotech). To differentiate naïve CD4^+^ T cells into pTh17 cells, the culture medium was enriched with 20 ng/mL IL-6 (Peprotech), 20 ng/mL IL-1β (Peprotech), and 20 ng/mL IL-23 (eBioscience). The cells were incubated at 37 ℃ in an atmosphere containing 5% CO_2_ for 72 h.

### RNA extraction and quantitative reverse transcription-PCR (qRT-PCR) analysis

We extracted total RNA using Trizol (Accurate, Changsha, Hunan, China). Following the manufacturer's guidelines, the HiScript Ⅱ Q RT SuperMix for qPCR (Vazyme, Nanjing, Jiangsu, China) was used to reverse-transcribe RNA into complementary deoxyribonucleic acid (cDNA). Quantitative PCR was conducted using ChamQ SYBR qPCR Master Mix (Vazyme) with gene-specific primers. The sequences of primer pairs are listed in the ***Supplementary Table 1*** (available online). Gene expression levels were standardized to *Actb*, and alterations in gene expression were determined using the 2^−ΔΔCT^ method.

### Protein extraction and Western blotting analysis

We lysed the harvested cells using lysis buffer on ice for half an hour, followed by a 10-minute boiling period. Protein concentration was determined using the bicinchoninic acid assay (Thermo Fisher, Waltham, Massachusetts, USA). Equal amounts of samples were separated by SDS-polyacrylamide gel electrophoresis and subsequently transferred to PVDF membranes. The membranes were then treated with 5% skim milk to block nonspecific binding, followed by incubation with primary and secondary antibodies sequentially. The PVDF membranes underwent scanning, followed by protein expression analysis with a gel imaging system. The primary antibodies used included anti-β-actin (1∶5000 dilution; Cat. #AF7018, Affinity, OH, USA), anti-FUNDC1 (1∶1000 dilution; Cat. #A22001, ABclonal, Wuhan, Hubei, China), anti-p-CaMKK2 (1∶1000 dilution; Cat. #AF4487, Affinity), and anti-p-AMPK (1∶1000 dilution; Cat. #ET1612-72, Huabio, Hangzhou, Zhejiang, China).

### Flow cytometry and intracellular cytokine staining

We performed surface staining by using anti-CD4 (BioLegend, San Diego, CA, USA) and anti-CD62L (BioLegend) for 30 min. For intracellular cytokine staining, cells were stimulated with 500 ng/mL phorbol 12-myristate 13-acetate (Sigma-Aldrich, Burlington, Vermont, USA), 500 ng/mL ionomycin (Sigma-Aldrich), and 10 μg/mL brefeldin A (eBioscience) for five to six hours. The cells were then treated with fixation/permeabilization buffer (eBioscience) in darkness at 4 ℃ for 50 min, followed by staining with specific antibodies such as anti-IL-17A (BioLegend), anti-IFN-γ (BioLegend), anti-RoRγt (BioLegend), and anti-IL-10 (eBioscience). The absolute number of cells was calculated based on the percentage of each population.

Following standard protocols, we treated the cells with tetramethylrhodamine ethyl ester (TMRE) probes (Beyotime, Shanghai, China), Fluo-4 AM probes (Beyotime), and MitoSOX Red mitochondrial superoxide indicator (Invitrogen, Carlsbad, CA, USA) to measure the mitochondrial membrane potential (MMP), intracellular Ca^2+^ levels, and mitochondrial reactive oxygen species (mROS), respectively.

### Mitochondrial fluorescent staining

Cells were collected in flow tubes and treated with 1× TMRE and 5 μmol/L MitoSOX probes, according to standard protocols for the detection of MMP or mROS, respectively. The cell nuclei were stained using Hoechst (Beyotime). After washing with culture medium, the cell suspension was applied to the slides, and the fluorescence intensity was examined by inverted fluorescence microscopy.

### Transmission electron microscopy

On the third day of naïve CD4^+^ T cell differentiation into pTh17 cells, we harvested the cells, centrifuged them, and preserved them in 0.1 mol/L PBS with 3% glutaraldehyde. Following sectioning and dual staining with uranium-lead, images were observed and recorded using a Carl Zeiss Gemini EM 10 CR electron microscope (ZEISS, Oberkochen, BW, Germany), and were evaluated by an independent investigator who was unaware of the study details.

### Mitochondrial stress test

We used a Seahorse XF extracellular flux analyzer (Agilent, Santa Clara, CA, USA) to measure the oxygen consumption rate in pTh17 cells. To immobilize the cells, 150000 cells per well were plated on XF96 microplates pre-treated with poly-D-lysine (Sigma). Before performing the assay, the cells were kept in XF medium (Seahorse Agilent) within a non-CO_2_ incubator for 20 min. The oxygen consumption rate was assessed using the Mito Stress Test Kit (Agilent) by sequentially administering 1 µmol/L oligomycin, 1.5 µmol/L FCCP, and 0.5 µmol/L rotenone/antimycin A. The data were analyzed using Wave software.

### PCR array

We used PCR array plates to examine gene expression profiles linked to mitochondrial energy metabolism, following the manufacturer's instructions (Wcgene Biotech, Shanghai, China). The data were analyzed using Wegene Biotech software. Genes exhibiting a fold change exceeding 2 or falling below −2 were considered biologically significant.

### RNA sequencing

RNA was extracted from activated splenic CD4^+^ T cells derived from *Adipor1* CKO and WT mice. cDNA sequencing libraries were generated and sequenced using the Illumina HiSeq platform, employing a 2 × 150 base pair paired-end sequencing approach. Differential gene expression was assessed by calculating fold changes in expression for each gene, determined by the ratio of the average fragments per kilobase of transcript per million mapped reads in the experimental group to that in the control group. The RNA sequencing (RNA-Seq) data were deposited in the NCBI Sequence Read Archive under the accession number PRJNA624232.

### Lentiviral transfection of T cells

Lentivirus expressing *Fundc1*-specific shRNA was constructed by GeneChem Co. Ltd. (Shanghai, China), and a lentivirus expressing only GFP was constructed as a control. The shRNA-*Fundc1* sequence was 5′-GAAGACACCACTGGTGGAATC-3′. Naïve CD4^+^ T cells were plated in 96-well plates at a density of 2 × 10^5^ cells per 200 μL and activated with anti-CD3e mAb and anti-CD28 mAb for 24 h. Lentiviral vectors were then added to the 96-well plates at a multiplicity of infection of 50. The medium was refreshed 24 h post-transfection. The transfection efficiency was evaluated by observing the cells through a fluorescence microscope, and it was also measured by qRT-PCR analysis.

### Statistical analysis

We performed statistical analyses using GraphPad Prism version 9.0.0 as well as one-way ANOVA and Student's *t*-tests. Significance was determined for *P*-values less than 0.05.

## Results

### *Adipor1* deficiency reduced pTh17 cell differentiation *in vitro*

Initially, we used magnetic beads to sort naïve CD4^+^ T cells from the spleens of WT mice (***Supplementary Fig. 1A***, available online). After inducing the differentiation of naïve CD4^+^ T cells into pTh17 and npTh17 cells for 72 h, we successfully generated CD4^+^IL-17A^+^ Th17 cells in both groups without a statistically significant difference (***Supplementary Fig. 1B***, available online). Flow cytometry showed minimal IL-10 protein levels in the pTh17 group, while the npTh17 group exhibited a significant increase in IL-10 protein levels (***Supplementary Fig. 1C***, available online). The qRT-PCR results showed increased *Csf2* and *Il23r* expression levels, but decreased *Il10* and *Cd5l* expression levels in pTh17 cells compared with npTh17 cells (***Supplementary Fig. 1D***, available online). We also examined the expression levels of other genes involved in the production of IL-10, including *Ikzf3*, *Ahr* and *Maf*, and found a significant difference in expression levels of *Ikzf3* between the groups (***Supplementary Fig. 1D***). These results indicate that pTh17 and npTh17 exhibit distinct inflammatory properties, which is consistent with the literature^[6–7,10–12]^, and suggest that our induced differentiation system is effective.

**Figure 1:**
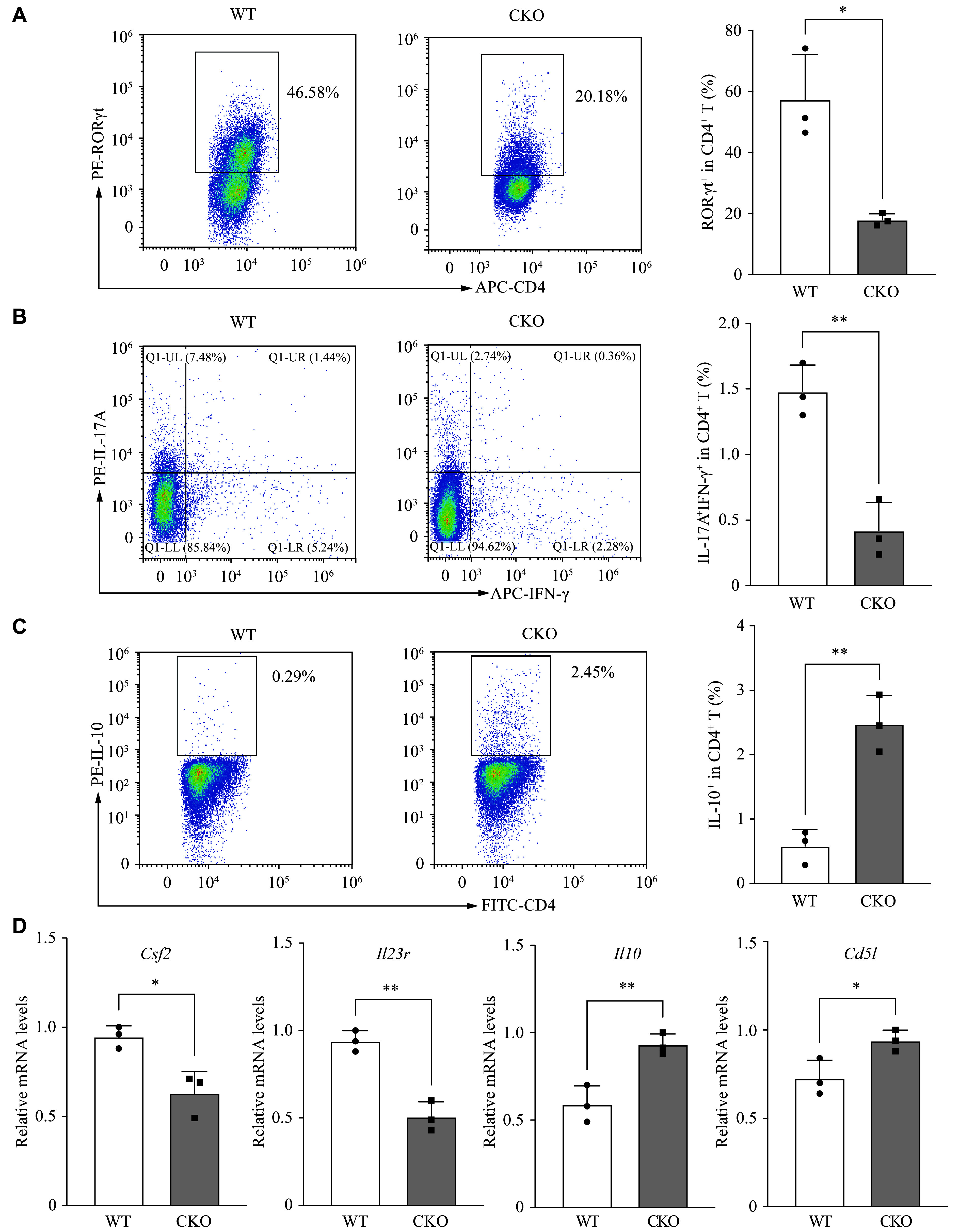
*Adipor1* knockout reduced pathogenic Th17 (pTh17) cell differentiation *in vitro*. Naïve CD4^+^ T cells from WT and *Adipor1* CKO mice were cultured under pTh17 differentiation conditions for three days. A–C: The percentages of CD4^+^RORγt^+^ live cells (A), pathogenic (IL-17A^+^IFN-γ^+^) Th17 cells (B), and CD4^+^IL-10^+^ live cells (C) were detected by flow cytometry (*n* = 3). D: Relative *Csf2*, *Il23r*, *Il10*, and *Cd5l* mRNA expression levels were detected by qRT-PCR (*n* = 3). Data are presented as mean ± standard deviation. Statistical analysis was performed using Student's *t*-test. ^*^P < 0.05 and ^**^P < 0.01. Abbreviations: WT, wild type; CKO, conditional knockout; qRT-PCR, quantitative reverse transcription-PCR.

Subsequently, the spleen-derived naïve CD4^+^ T cells from both *Adipor1* CKO and WT mice were induced to differentiate into pTh17 cells for 72 h. Given that pTh17 cells possess the signature of Th17 cells, we initially examined the expression of RORγt, a transcription factor characteristic of these cells. A significant reduction in CD4^+^RORγt^+^ T cells was observed in the *Adipor1* CKO group compared with the WT group (***Fig. 1A***). Because Th17 cells are pathogenic when TNF-α, IFN-γ, and GM-CSF are produced simultaneously, along with IL-17A^[6]^, we investigated whether AdipoR1 regulated the differentiation of pTh17 (IL-17A^+^IFN-γ^+^) cells. The results showed that *Adipor1* knockout significantly reduced the proportion of pTh17 cells (***Fig. 1B***). We also examined the characteristic genes associated with pTh17 and npTh17 cells. Flow cytometry showed that IL-10 protein levels were significantly elevated in pTh17 cells from the *Adipor1* CKO group, compared with those from the WT group (***Fig. 1C***). Meanwhile, qRT-PCR analysis revealed that pTh17 cells in the *Adipor1* CKO group had lower expression levels of the pro-inflammatory molecules *Csf2* and *Il23r*, but higher expression levels of *Il10* and *Cd5l* than those in the WT group (***Fig. 1D***). These results suggest that *Adipor1* knockout may inhibit pTh17 differentiation.

### *Adipor1* deficiency impaired mitochondrial function in pTh17 cells

**Figure 2:**
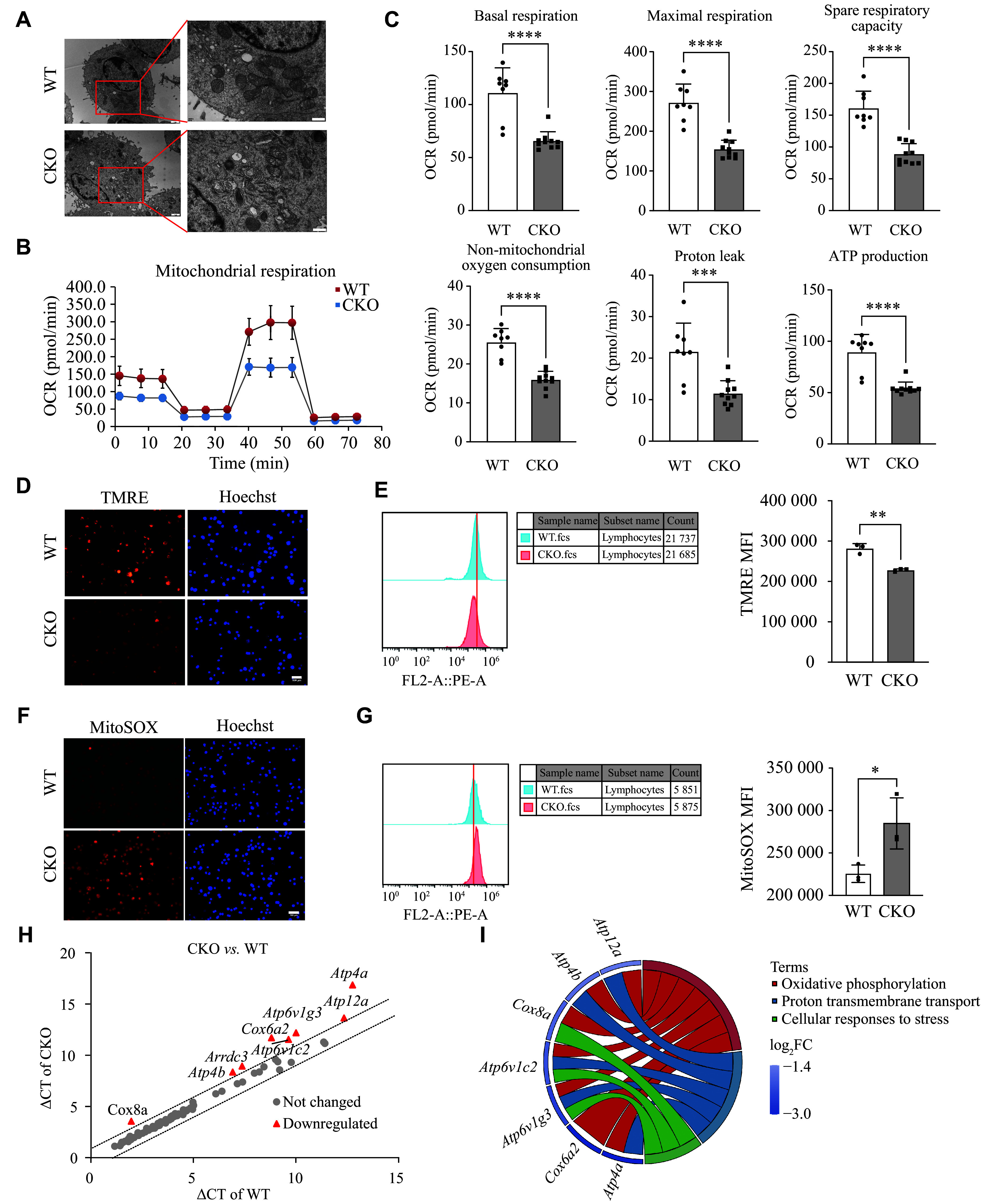
*Adipor1* knockout inhibited mitochondrial function in pathogenic Th17 (pTh17) cells. Naïve CD4^+^ T cells from WT and *Adipor1* CKO mice were cultured under pTh17 differentiation conditions for three days. A: Images of pTh17 cells were captured using transmission electron microscopy. Left: representative images at 12000× magnification; scale bar, 1 μm. Right: enlarged area of the red box at 30000× magnification; scale bar, 500 nm (*n* = 3). B: Mitochondrial stress test curve of pTh17 cells (*n* = 8–10). C: Basal respiration, ATP-associated respiration, proton leakage, maximal respiration, spare respiratory capacity, and non-mitochondrial oxygen consumption of pTh17 cells (*n* = 8–10). D and E: Mitochondrial membrane potential was measured by using TMRE probes, with fluorescence observed using a microscope (D; Scale bar, 100 μm) and the MFI of TMRE quantified by flow cytometry (E) (*n* = 3). F and G: ROS levels in mitochondria were measured by using MitoSOX probes, with fluorescence observed using a microscope (F; Scale bar, 100 μm) and the MFI of MitoSOX quantified by flow cytometry (G). H and I: Detection of 90 key genes related to mitochondrial energy metabolism using PCR arrays. Scatter plots of differentially expressed genes in pTh17 cells (H) and enrichment analysis of the differentially expressed genes (I). Data are presented as mean ± standard deviation. Statistical analysis was performed using Student's *t*-test. ^*^P < 0.05, ^**^P < 0.01, ^***^P < 0.001, and ^****^P < 0.0001. Abbreviations: WT, wild type; CKO, conditional knockout; MMP, mitochondrial membrane potential; TMRE, tetramethylrhodamine ethyl ester; MFI, mean fluorescence intensity; ROS, reactive oxygen species.

Considering that the mitochondrial shape and cristae architecture influence their functions, we initially employed transmission electron microscopy to observe the changes in mitochondrial morphology in pTh17 cells. We observed that mitochondria in pTh17 cells from the *Adipor1* CKO group appeared light in color, with pronounced vacuolization and blurred cristae, compared with those in the WT group (***Fig. 2A***).

Subsequently, we assessed mitochondrial respiration in pTh17 cells to investigate the role of AdipoR1 in mitochondrial function. The results showed that mitochondrial respiration, as indicated by oxygen consumption rates, was significantly lower in pTh17 cells from the *Adipor1* CKO group than in those from the WT group (***Fig. 2B*** and ***2C***). We also measured the MMP using TMRE probes and found that *Adipor1* knockout significantly reduced the MMP of pTh17 cells (***Fig. 2D*** and ***2E***). In addition to ATP production, another crucial function of mitochondria is the generation of mROS. We observed that *Adipor1* knockout induced a significant increase in mROS in pTh17 cells (***Fig. 2F*** and ***2G***).

Furthermore, we performed PCR arrays to examine the genes involved in mitochondrial energy metabolism. The results showed that the mRNA levels of *Atp4a*, *Cox6a2*, *Atp6v1g3*, *Atp6v1c2*, *Cox8a*, *Arrdc3*, *Atp4b*, and *Atp12a* were significantly reduced in pTh17 cells from the *Adipor1* CKO group, compared with those from the WT group (***Fig. 2H***). These differentially expressed genes primarily affect proton transmembrane transport and oxidative phosphorylation levels (***Fig. 2I***). All these results indicate that *Adipor1* knockout may inhibit the mitochondrial functions of pTh17 cells.

### RNA-Seq screened for differentially expressed gene *Fundc1* in the activated CD4^+^ T cells derived from *Adipor1* CKO and WT mice

**Figure 3:**
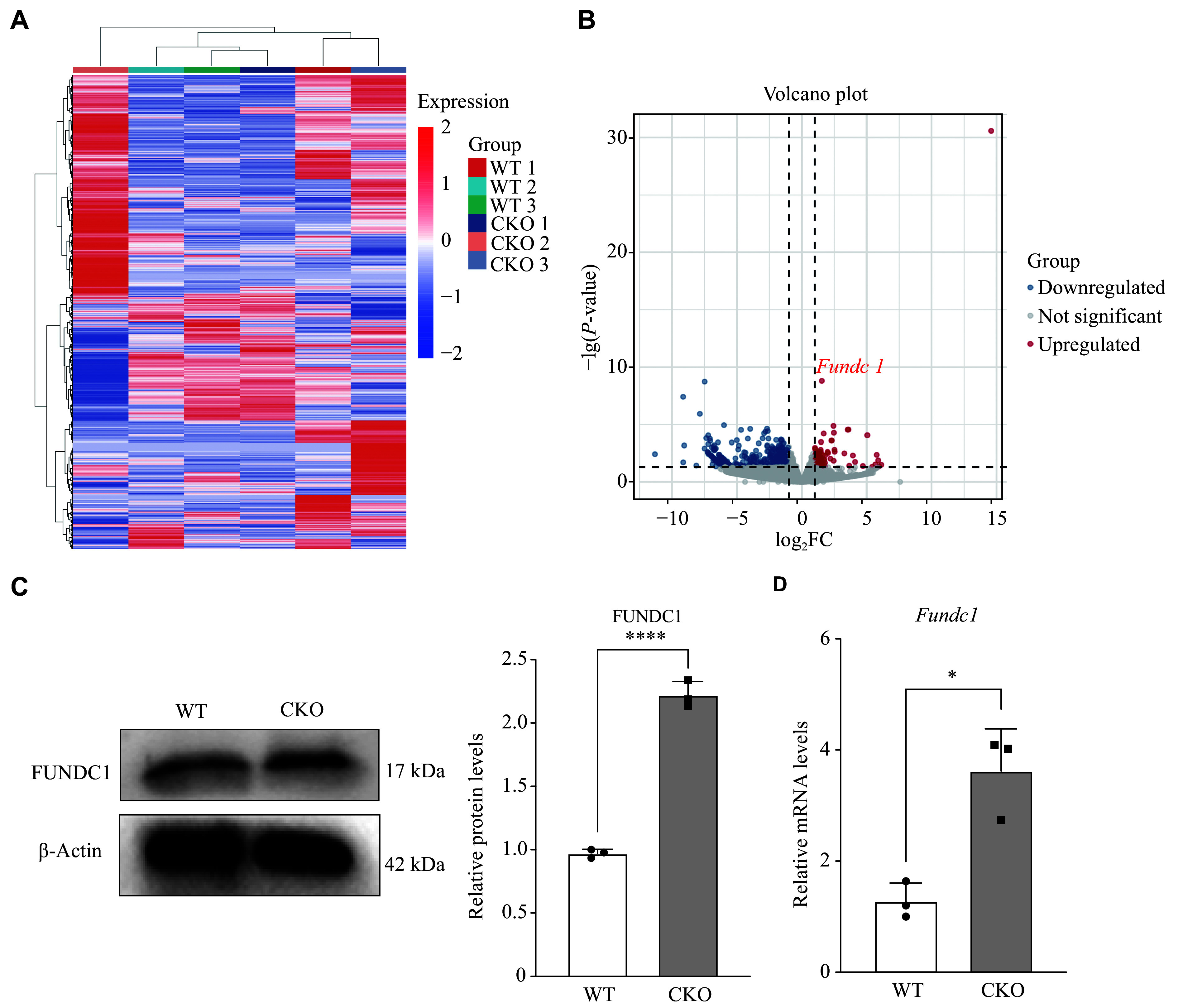
RNA-sequencing analysis of activated CD4^+^ T cells in WT and *Adipor1* CKO mice. A: Heatmap of 646 differentially expressed genes in activated CD4^+^ T cells from the WT and *Adipor1* CKO mice. Columns display mouse samples, while rows highlight genes with differential expression. Red indicates increased expression, and blue indicates decreased expression. B: Volcano plot analysis of 646 differentially expressed genes. Red indicates increased expression, and blue indicates decreased expression. C and D: Protein (C) and mRNA (D) levels of *Fundc1* in pathogenic Th17 cells from the WT and *Adipor1* CKO mice were analyzed by Western blotting and qRT-PCR, respectively (*n* = 3). Data are presented as mean ± standard deviation. Statistical analysis was performed using Student's *t*-test. ^*^P < 0.05 and ^****^P < 0.0001. Abbreviations: WT, wild type; CKO, conditional knockout; qRT-PCR, quantitative reverse transcription-PCR; FC, fold change.

We analyzed gene expression profiles of the activated CD4^+^ T cells derived from WT and *Adipor1* CKO mice by RNA-Seq, and identified 95 upregulated and 551 downregulated genes in the *Adipor1* CKO group, with *Fundc1* being the most significantly altered gene (*P* = 1.56 × 10^−9^, log_2_FC = 1.54; ***Fig. 3A*** and ***3B***). We further examined the expression of *Fundc1* by Western blotting and qRT-PCR in pTh17 cells. The results showed that both protein and mRNA expression levels of *Fundc1* were significantly increased in pTh17 cells from *Adipor1* CKO mice, compared with those from WT mice (***Fig. 3C*** and ***3D***), which was consistent with the RNA-Seq results.

### Effect of *Adipor1* deficiency on intracellular Ca^2+^ levels and upstream target gene of FUNDC1 in pTh17 cells

**Figure 4:**
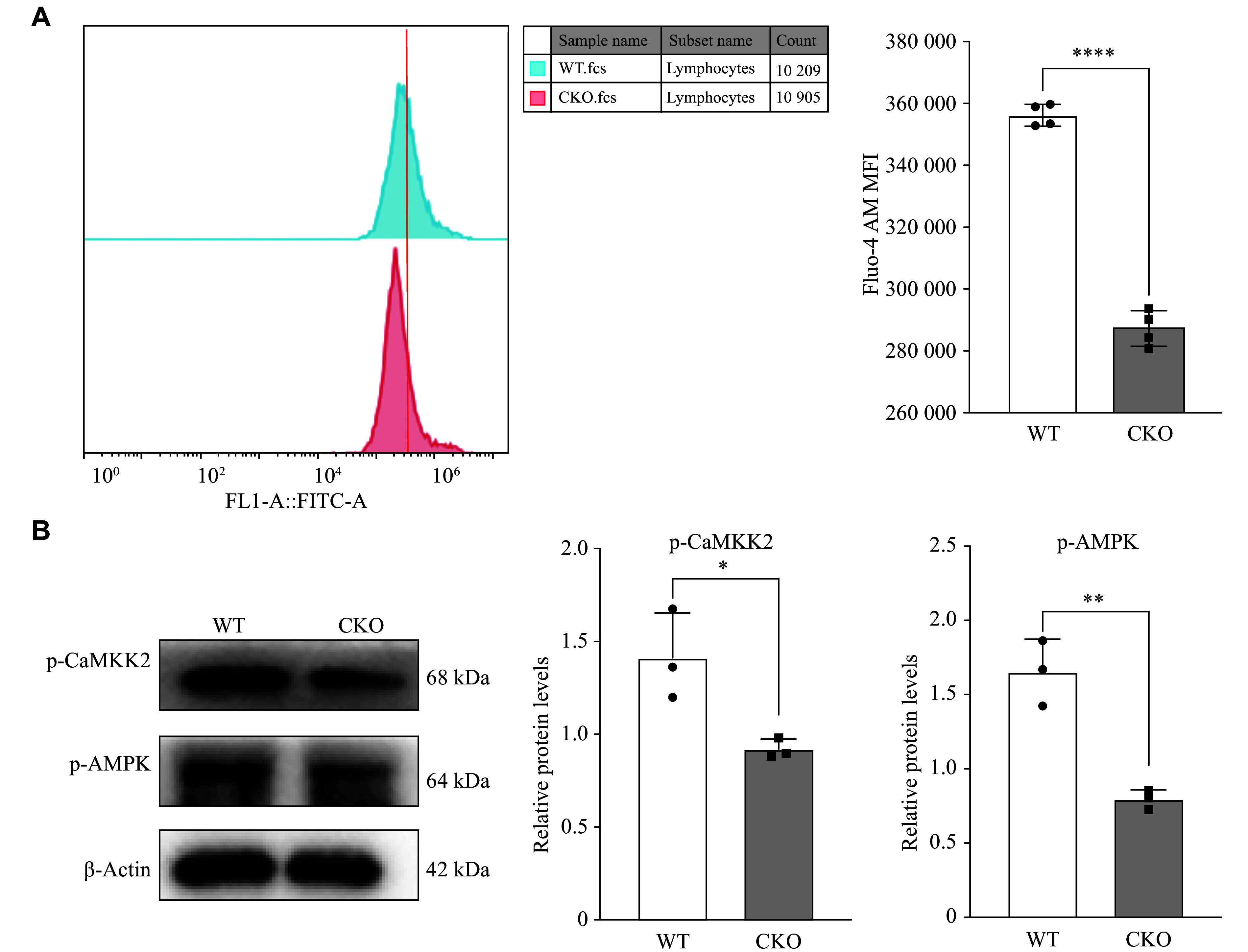
Effect of *Adipor1* knockout on intracellular Ca^2+^ concentration and downstream target genes in pathogenic Th17 (pTh17) cells. A: The intracellular Ca^2+^ concentration, indicated by the Fluo-4 AM calcium ion fluorescent probe, was assessed through flow cytometry and quantified using the MFI of Fluo-4 AM (*n* = 3). B: Western blot analysis was performed to examine the protein levels of p-CaMKK2 and p-AMPK in pTh17 cells (*n* = 3). Data are presented as mean ± standard deviation. Statistical analysis was performed using Student's *t*-test. ^*^P < 0.05, ^**^P < 0.01, and ^****^P < 0.0001. Abbreviation: MFI, mean fluorescence intensity.

Adiponectin was reported to increase intracellular Ca^2+^ concentration by inducing extracellular Ca^2+^ influx *via* AdipoR1, and this intracellular Ca^2+^ accumulation activated the CaMKK2/AMPK signaling cascade, with FUNDC1 identified as a downstream target of AMPK^[20–22]^. Therefore, we used Fluo-4 AM calcium ion fluorescent probes to examine the intracellular Ca^2+^ concentration and found that the intracellular Ca^2+^ levels were significantly reduced in pTh17 cells from *Adipor1* CKO mice, compared with those from WT mice (***Fig. 4A***). Additionally, Western blotting showed that the phosphorylation levels of both CaMKK2 and its downstream kinase AMPK in pTh17 cells from the *Adipor1* CKO group were significantly lower than those from the WT group (***Fig. 4B***). These results suggest that AdipoR1 may inhibit FUNDC1 expression by activating AMPK phosphorylation through the regulation of transient calcium influx.

### Inhibition of FUNDC1 attenuated the effect of *Adipor1* deficiency on pTh17 cell differentiation and mitochondrial function

**Figure 5:**
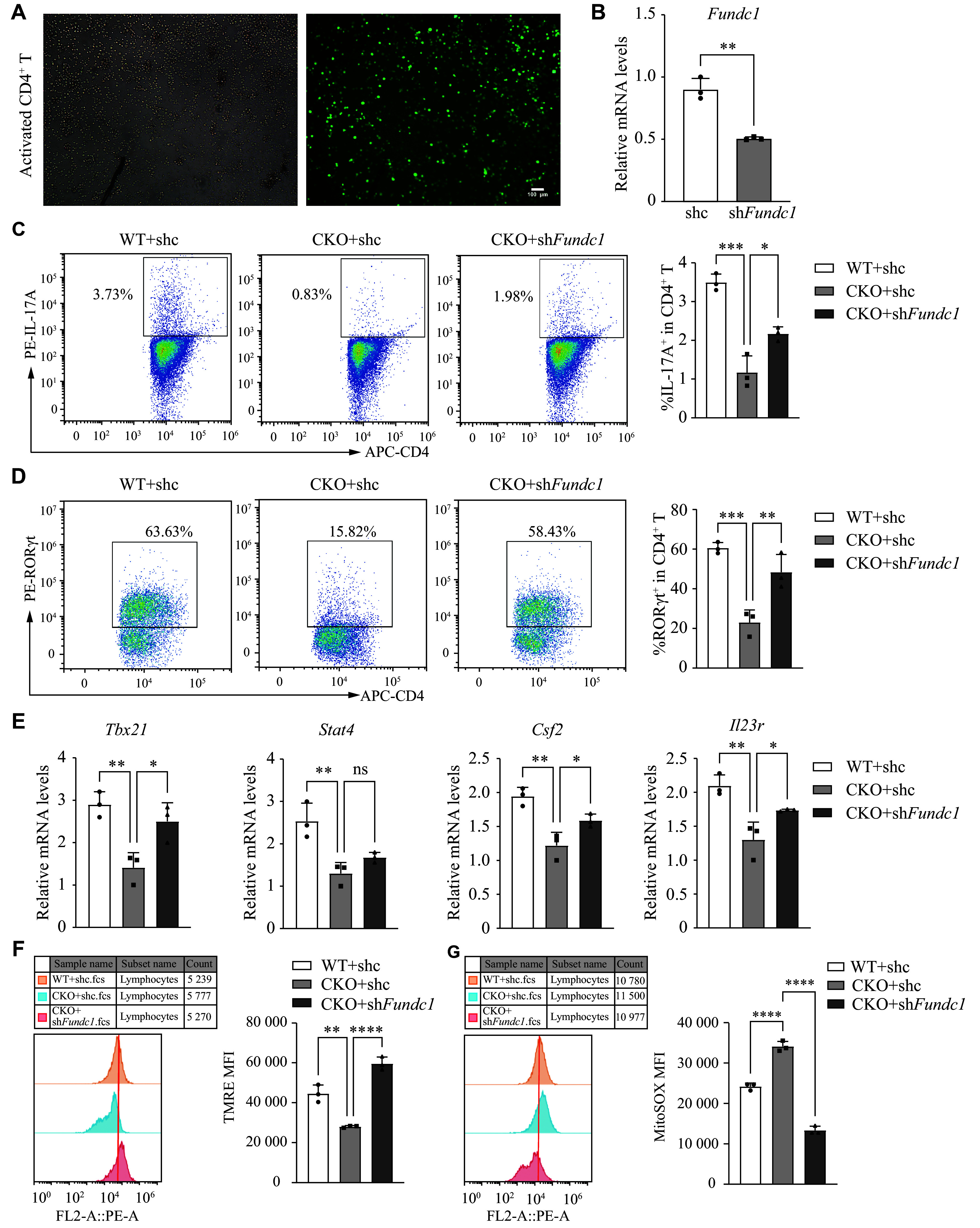
*Fundc1* knockdown attenuated the effects of *Adipor1* knockout on pTh17 cell differentiation and mitochondrial function. Naïve CD4^+^ T cells were first activated for 24 h, followed by infection with lentiviruses expressing shRNA targeting *Fundc1* (sh*Fundc1*) or lentiviral vectors containing only GFP (shc) for 16 h, and then were cultured under pTh17 differentiation conditions for 72 h. A: Fluorescence microscopy was used to observe CD4^+^ T cells infected with sh*Fundc1* lentivirus; Scale bar, 100 μm. B: The knockdown efficiency of *Fundc1* was detected by qRT-PCR (*n* = 3). C and D: The percentages of CD4^+^IL-17A^+^ (C) and CD4^+^Rorγt^+^ (D) live cells were detected by flow cytometry (*n* = 3). E: *Tbx21*, *Stat4*, *Csf2*, and *Il23r* mRNA levels were detected by qRT-PCR (*n* = 3). F: Mitochondrial membrane potential was measured by using TMRE probes, and the MFI of TMRE was detected by flow cytometry (*n* = 3). G: ROS levels in mitochondria were measured by using MitoSOX probes, and the MFI of MitoSOX was quantified by flow cytometry (*n* = 3). Data are presented as mean ± standard deviation. Statistical analysis was performed using Student's *t*-test (B) and one-way ANOVA (C–E). ^*^P < 0.05,^**^P < 0.01, ^***^P < 0.001, and ^****^P < 0.0001. Abbreviations: qRT-PCR, quantitative reverse transcription-PCR; MFI, mean fluorescence intensity; TMRE, tetramethylrhodamine ethyl ester; ns, not significant.

To investigate whether AdipoR1 regulates pTh17 differentiation through FUNDC1, we infected activated naïve CD4^+^ T cells with lentiviruses expressing *Fundc1*-specific shRNA (***Fig. 5A***). The mRNA expression of *Fundc1* was significantly reduced following sh*Fundc1* lentivirus infection, with a mean decrease of approximately 50% (***Fig. 5B***). Under Th17 cell differentiation conditions, *Fundc1* knockdown not only significantly restored the reduction in IL-17A and RORγt expression induced by *Adipor1* deficiency (***Fig. 5C*** and ***5D***), but also partially restored the expression of cytokines and transcription factors associated with pTh17 cells (***Fig. 5E***). To further investigate the effect of *Fundc1* knockdown on mitochondrial function in pTh17 cells, we examined the MMP and mROS levels. The results showed that *Fundc1* knockdown significantly attenuated the decrease in MMP and the accumulation of mROS induced by *Adipor1* deficiency (***Fig. 5F*** and ***5G***). These findings indicate that AdipoR1 may affect mitochondrial function *via* FUNDC1 to enhance the differentiation of pTh17 cells.

## Discussion

pTh17 cells are thought to play a critical role in the pathogenesis of rheumatoid arthritis, which may be diagnosed, treated, and prognosed by targeting pTh17 cells or their associated molecules^[25]^. In the present study, we demonstrated that *Adipor1* knockout significantly reduced the proportion of naïve CD4^+^ T cells differentiating into pTh17 cells. We also found that the loss of *Adipor1* in pTh17 cells resulted in gene expression profiles similar to those observed in WT npTh17 cells. The results indicate that AdipoR1 is essential for the development and activity of pTh17 cells, and its absence skews these cells towards an npTh17-like phenotype. This highlights the potential of targeting AdipoR1 to modulate pTh17 cell activity in treatment strategies for rheumatoid arthritis.

The role of adiponectin and its receptors in Th17 cell differentiation is currently controversial, possibly because adiponectin exists in the plasma as multimeric forms, including trimer, hexamer, high-molecular-weight oligomer, and a free globular structural domain generated by C-terminal proteolytic cleavage, which leads to different biological functions^[26–27]^. Adiponectin exerts its biological function through its receptors, AdipoR1 and AdipoR2. AdipoR1 is highly expressed in skeletal muscle, whereas AdipoR2 is highly expressed in the liver and kidneys. Both receptors belong to the G protein-coupled receptor family but differ in amino acid sequence, resulting in distinct affinities and binding specificities for adiponectin. For instance, KS23, a novel adiponectin-derived peptide, has been shown to downregulate the proportion of Th17 cells in experimental autoimmune uveitis^[28]^. This appears to contradict our findings, but it is unclear whether KS23 acts directly on AdipoR1. It has also been reported that AdipoRon, an agonist of AdipoR1 and AdipoR2, inhibits Th17 cell differentiation. However, the effect of AdipoRon on Th17 cell differentiation was attenuated by AdipoR2 blockers but not by AdipoR1 blockers^[29]^. Therefore, we tested the effect of *Adipor1* deficiency on AdipoR2 expression and found a tendency for AdipoR2 to be reduced at the protein and nucleic acid levels, but there was no statistically significant difference (***Supplementary Fig. 2***, available online). Several studies are consistent with our findings^[27]^. Thus, these receptors play different roles under different physiological and pathological conditions.

Additionally, we found that *Adipor1*-deficient pTh17 cells had abnormal mitochondrial morphology and impaired mitochondrial function. To investigate the potential mechanisms by which AdipoR1 regulates pTh17 cell differentiation, we analyzed the gene expression profiles of the activated CD4^+^ T cells derived from both *Adipor1* CKO and WT mice. This analysis identified *Fundc1* as a significantly differentially expressed gene, which was validated in pTh17 cells with *Adipor1* knockout. FUNDC1, a mammalian mitophagy receptor, interacts with and recruits microtubule-associated protein 1A/1B-light chain 3 (LC3) to mitochondria for mitophagy^[21]^. In contrast, in mammals, phosphorylated FUNDC1 inhibits binding to LC3, thereby blocking subsequent mitophagy^[30–31]^. FUNDC1 is also a novel MAM protein that is enriched at the MAM. In cardiomyocytes from a mouse model of diabetes, FUNDC1 was highly expressed and might promote MAM formation by interacting with IP3R2, thereby increasing Ca^2+ ^and FIS1 expression in mitochondria, ultimately leading to mitochondrial dysfunction and impaired myocardial structure and function^[32]^. These findings highlight the crucial role of FUNDC1 in regulating mitochondrial function. Therefore, we hypothesized that *Adipor1* knockout might inhibit pTh17 cell differentiation by affecting mitochondrial function through the upregulation of FUNDC1 expression.

AdipoR1, as a membrane protein, regulates FUNDC1 expression by inducing extracellular Ca^2+^ influx, thereby increasing intracellular Ca^2+^ concentrations^[33]^. This intracellular Ca^2+^ accumulation activates the CaMKK2/AMPK signaling cascade^[33–34]^. Specific disruption of AdipoR1 suppresses adiponectin-mediated increases in intracellular Ca^2+^ concentration and reduces CaMKK and AMPK activation^[33]^. Research has shown that AMPK activation-mediated inhibition of FUNDC1 is an effective approach to treating diabetic cardiomyopathy, confirming FUNDC1 as a downstream target of AMPK^[32]^. We detected intracellular Ca^2+^ levels by flow cytometry and analyzed the CaMKK2 and AMPK phosphorylation levels by Western blotting. Our results suggest that AdipoR1 may inhibit FUNDC1 expression by activating AMPK phosphorylation through the regulation of transient calcium influx.

To investigate further, we constructed lentiviruses to interfere with FUNDC1 expression and observed whether the effects of *Adipor1* knockout on mitochondrial function and pTh17 differentiation could be reversed. The results showed that *Fundc1* knockdown partially restored both mitochondrial function and the differentiation capacity of *Adipor1*-deficient pTh17 cells. However, the precise mechanisms by which FUNDC1 affects mitochondrial function require further experimental validation.

In summary, this research is the first to demonstrate that *Adipor1* knockout upregulates FUNDC1 expression, thereby inhibiting pTh17 differentiation through mitochondrial dysfunction. Our findings establish a novel molecular mechanism by which AdipoR1 regulates the pTh17 pathway to affect rheumatoid arthritis as well as other autoimmune and inflammatory diseases mediated by pTh17. These findings offer a conceptual framework and empirical support for developing novel therapeutic strategies for these diseases.

## SUPPLEMENTARY DATA

Supplementary data to this article can be found online.
