# Supplementary material for: AdipoR1 promotes pathogenic Th17 differentiation by regulating mitochondrial function through FUNDC1
Source: J Biomed Res. 2024 Nov 7;39(3):305–16. doi: 10.7555/JBR.38.20240244 (PMC12239983; doi:10.7555/JBR.38.20240244)
Supplement: Supplementary file 1 — Supplementary data to this article can be found online. [file jbr-39-3-305-Supplementary.pdf]

Available online at www.jbr-pub.org.cn

Open Access at PubMed Central

Journal of Biomedical Research, 2025 39(3): 1–3

Supplementary Data

JBR

AdipoR1 promotes pathogenic Th17 differentiation by regulating mitochondrial function through FUNDC1

Hui Wang△, Qian Zhang△, Yuankai Sun, Wenfeng Tan✉, Miaojia Zhang✉

Department of Rheumatology, the First Affiliated Hospital of Nanjing Medical University, Nanjing, Jiangsu 210029, China.

Supplementary Table 1 **Primers for real-time quantitative PCR analysis**

Genes	Primer sequences (5′-3′)
Actb	Forward: GACAGGATGCAGAAGGAGA<br>Reverse: GTACTTGCGCTCAGGAGG AG
Adipor1	Forward: AACTGGACTATTCAGGGATTGC<br>Reverse: ACCATAGAAGTGGACGAAAGC
Csf2	Forward: GGCCTTGGAAGCATGTAGAGG<br>Reverse: GGAGAACTCGTTAGAGACGACTT
Il23r	Forward: AACAACAGCTCGGATTTGGTAT<br>Reverse: ATGACCAGGACATTCAGCAGT
Il10	Forward: CTTACTGACTGGCATGAGGATCA<br>Reverse: GCAGCTCTAGGAGCATGTGG
Cd5l	Forward: CAGGCTGGAACTTACAG GTCT<br>Reverse: GTCCTTTGCCCTGAGTATTCTTG
Ikzf3	Forward: GGAGCTGAAAAGCACGGAGG<br>Reverse: TCGAATCTTCTCCTGCATCTTCGT
Ahr	Forward: TCTTT GATGGCGCTGAATGG<br>Reverse: ACTGCTGAAAGCCCAGGTAAT
Maf	Forward: AGGATGGCTTCAGAACTGGC<br>Reverse: GGTCTCCACCGGTTCCTTTT
Il17a	Forward: CTCCAGAAGGCCCTCAGACTAC<br>Reverse: AGCTTTCCCTCCGCATTGACACAG
Rorc	Forward: TCCACTACGGGGTTATCACCT<br>Reverse: AGTAGGCCACATT ACACTGCT
Fundc1	Forward: AGCGATGACGAATCATACGAAG<br>Reverse: CCAC CCATTACAATCTGAGTAGC
Stat4	Forward: GCAGCCAACATGCCTATCCA<br>Reverse: TGGCAGACACTTTGTGTTCCA
Tbx21	Forward: AGCAAGGACGGCGAATGTT<br>Reverse: GTGGACATATAAGCGGTTCCC

△These authors contributed equally to this work.

✉Corresponding authors: Wenfeng Tan and Miaojia Zhang, Department of Rheumatology, the First Affiliated Hospital of Nanjing Medical University, 300 Guangzhou Road, Nanjing, Jiangsu 210029, China. E-mails: tw2006@njmu.edu.cn (Tan) and miaojia_zhang@163.com (Zhang).

Received: 08 August 2024; Revised: 23 October 2024; Accepted: 26 October 2024; Published online: 07 November 2024

CLC number: R392.12, Document code: A

The authors reported no conflict of interests.

This is an open access article under the Creative Commons Attribution (CC BY 4.0) license, which permits others to distribute, remix, adapt and build upon this work, for commercial use, provided the original work is properly cited.

© 2025 by Journal of Biomedical Research.

https://doi.org/10.7555/JBR.38.20240244

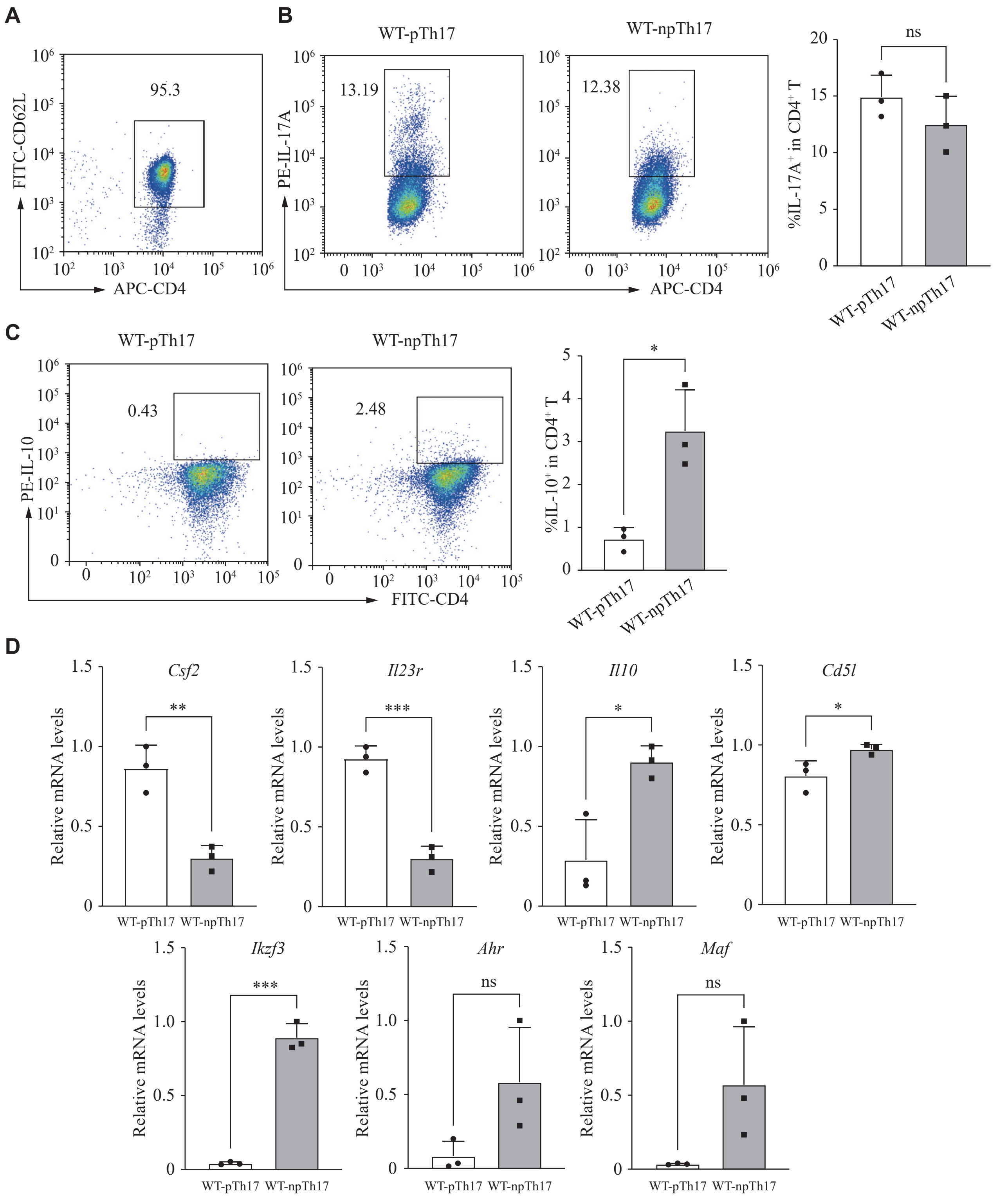

Supplementary Fig. 1 **Induction of differentiation and characterization of pTh17 and npTh17.** A: Representative flow cytometry plots of naïve CD4+ T cells are shown. B and C: Naïve T cells from WT mice were cultured under pTh17 and npTh17 differentiation conditions, respectively. Representative flow cytometry plots of CD4+IL-17A+ (B) and CD4+IL-10+ (C) live cell populations are shown (n = 3). D: Relative *Csf2*, *Il23r*, *IL-10*, *Cd5l*, *Ikzf3*, *Ahr*, and *Maf* mRNA expression levels were examined by qRT-PCR (n = 3). Data are presented as mean ± standard deviation. Statistical analysis was performed using Student's t-test. $^{*}P < 0.05$, $^{**}P < 0.01$, and $^{***}P < 0.001$. Abbreviations: WT, wild type; qRT-PCR, quantitative reverse transcription-PCR; ns, not significant.

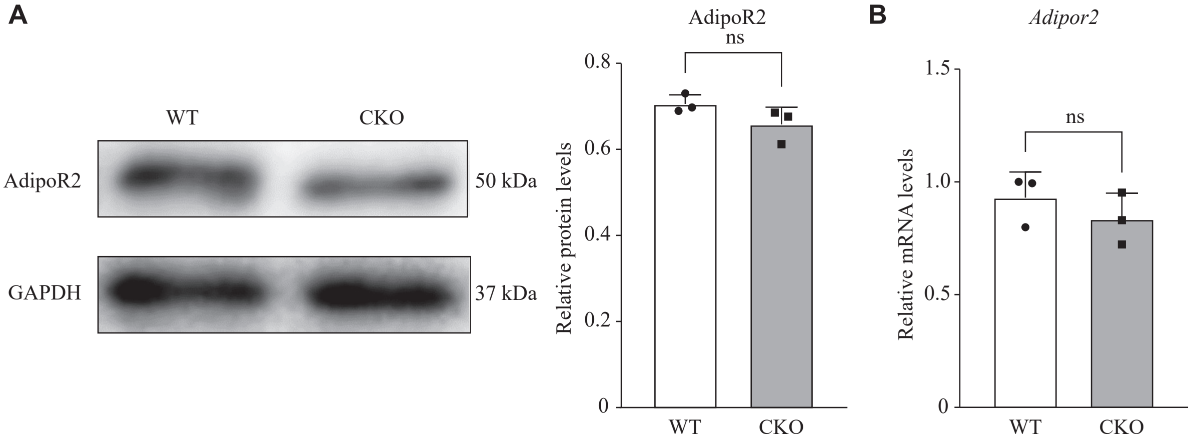

Supplementary Fig. 2 **Effect of *Adipor1* deficiency on AdipoR2 expression.** A: Western blotting analysis was used to examine AdipoR2 protein levels in CD4+ T cells of *Adipor1*-deficient mice (n = 3). B: *Adipor2* mRNA levels in CD4+ T cells were measured by qRT-PCR (n = 3). Data are presented as mean ± standard deviation. Statistical analysis was performed using Student's t-test. Abbreviations: qRT-PCR, quantitative reverse transcription-PCR; ns, not significant.
